# Residential Mobility, Housing Instability, Adverse Childhood Experiences, and the Moderating Role of Neighborhood Contexts

**DOI:** 10.3390/ijerph23030326

**Published:** 2026-03-06

**Authors:** Jaeyong Yoo, Satya Fisher, Jaehwan Kim

**Affiliations:** 1 Property Management Program, Virginia Tech, Blacksburg, VA 24061, USA; 2 Real Estate Development, Habitat for Humanity, New York, NY 10038, USA; sfisher@habitatnycwc.org; 3 Department of Real Estate, Kongju National University, Yesan 32588, Republic of Korea; jaehwan@kongju.ac.kr

**Keywords:** housing instability, residential mobility, adverse childhood experiences (ACEs), neighborhood effects, frequent moves, child health

## Abstract

**Highlights:**

**Public health relevance—How does this work relate to a public health issue?**

- Housing instability, measured by frequent residential moves, is closely linked to children’s exposure to adverse childhood experiences, a well-established determinant of long-term physical and mental health.
- By using nationally representative data, this study connects residential mobility to population-level patterns of childhood adversity in the United States.

**Public health significance—Why is this work of significance to public health?**

- The findings show that frequent residential moves more than double children’s risk of experiencing adverse childhood experiences, highlighting housing instability as a major but underrecognized public health risk factor.
- Neighborhood conditions and access to social supports shape how housing instability translates into childhood adversity, underscoring the importance of place-based health determinants.

**Public health implications—What are the key implications or messages for practitioners, policy makers and/or researchers in public health?**

- Public health screening and prevention efforts should incorporate housing instability indicators, such as frequent moves, to better identify children at heightened risk of adversity.
- Policies that reduce forced mobility and strengthen neighborhood supports, including rental assistance and community investment, may yield substantial public health benefits for children.

**Abstract:**

Housing instability, particularly frequent residential moves, has been associated with poor developmental outcomes, yet its relationship with adverse childhood experiences (ACEs) remains insufficiently understood at the national level. This study addresses this gap by investigating how frequent moves shape children’s exposure to ACEs, and whether community and household contexts influence these effects. Using the 2020–2021 National Survey of Children’s Health data, we ask two questions: (1) Do children who experience frequent moves face greater risk of ACEs? and (2) Do neighborhood and metropolitan contexts mitigate or exacerbate this association? Our contribution is twofold. First, we examine both directions of the relationship: how ACEs predict frequent moves and how frequent moves increase ACE exposure. Second, we incorporate contextual moderators, including supportive neighborhoods, safety, amenities, and urban residence, to provide a more nuanced account of how environments shape resilience or vulnerability. Using logistic and negative binomial regression models, we find that all ACEs significantly predict frequent moves, with parental divorce/separation showing the largest effect. Economic hardship is also a strong predictor of frequent residential mobility, and while food or cash assistance is associated with higher mobility, it moderates the hardship-mobility association. Supportive neighborhoods are associated with lower odds of moving. In turn, frequent moves more than double children’s risk of ACEs. Supportive and safe neighborhoods provide protective benefits, while detracting elements exacerbate adversity. We conclude that reducing frequent moves and strengthening neighborhood supports are critical strategies for mitigating childhood adversity.

## 1. Introduction

Housing instability can manifest in many forms, such as difficulties paying rent, overcrowding, frequent moves, or challenges in covering other basic needs [1]. In recent years, rising housing costs have intensified these pressures. Between the first quarter of 2020 and the first quarter of 2023, rents and home prices increased by 23.9 percent, making affordable housing increasingly difficult to secure, particularly in urban areas. Consequently, the share of households spending more than 30% of their income on housing costs reached historic highs in 2021 [2]. Such cost burdens leave families with little money to meet necessities like food, health care, and other living expenses [3,4,5]. To manage high housing costs, some households relocate to lower-rent neighborhoods that may also have higher crime rates or fewer resources [6]. Such moves can increase exposure to safety risks and reflect broader housing precarity, which is associated with a heightened risk of housing instability and forced displacement in some cases [7].

Residential relocation can be stressful for children even when moves are planned, as relocation may disrupt routines, schooling, and peer or community ties. However, moves that are involuntary or financially driven, such as those triggered by unaffordable housing costs, eviction threats, or family disruption, are more likely to be abrupt and destabilizing, and therefore more strongly associated with exposure to adverse childhood experiences (ACEs) than moves related to opportunity or life-course transitions [8].

Beyond financial hardship, housing instability has cascading consequences for health and well-being, particularly for families with children. A growing body of research demonstrates that housing instability is associated with poorer physical and mental health outcomes, while improvements in housing stability and quality are linked to reduced health risks [9,10,11,12]. These consequences are especially acute in childhood [13,14], when stable home environments provide a critical foundation for healthy development. Secure and supportive housing facilitates access to healthcare, education, and other essential services, whereas frequent moves disrupt routines and increase the risk of chronic conditions and poorer physical health outcomes, with implications that extend into adulthood [15].

Because children’s development occurs within broader community contexts, unstable housing also weakens ties to neighborhoods. Frequent moves can prevent families from forming lasting attachments to local communities, where neighborhood characteristics, such as safety, access to services, and crime levels, are closely linked to health outcomes [6]. Disruptions to these connections may further compound the risks associated with residential instability.

Despite the importance of these connections, the pathways linking chronic housing instability to ACEs remain inadequately understood, largely due to data limitations. Prior research has shown that children in unstable housing face an elevated risk of ACEs, which are robust predictors of poor health later in life [16]. Yet the mechanisms remain underexplored, and more evidence is needed to clarify how instability translates into long-term disadvantage.

Although emerging studies suggest that severe housing cost burdens are associated with higher odds of experiencing ACEs [17] and that instability should be accounted for when constructing ACE scales [18], existing work has important limitations. Most research has treated either housing instability or ACEs as an outcome, without fully considering their potential bidirectional relationship. Furthermore, little is known about how neighborhood and community contexts might shape or influence these dynamics.

This study addresses these gaps by examining the bidirectional relationship between housing instability and ACEs. We test both whether adverse experiences predict children’s likelihood of frequent moves and whether frequent moves, in turn, increase exposure to ACEs. In addition, we incorporate contextual moderators, assessing whether metropolitan residence, safe and supportive neighborhoods, neighborhood amenities, and detracting elements mitigate or exacerbate these associations. By integrating bidirectional analysis with contextual moderators, this study provides new insights into the mechanisms between instability and adversity, with implications for clinical practice and public policy aimed at reducing long-term health disparities.

## 2. Literature Review

### 2.1. What Is Housing Instability?

Housing instability is commonly understood as a condition in which households lack sufficient control over their residential environments [19]. It can take many forms, including frequent moves, evictions, overcrowding, or doubling up with non-family members [1]. These circumstances often arise when families are burdened by high housing costs [20,21].

Housing instability is shaped by interacting economic, policy, and social factors. In addition to affordability pressures, social and household conditions, such as overcrowding, frequent relocations, and living arrangements involving non-family members, often reflect underlying economic stress, family disruption, or constrained housing options. These social dimensions of instability frequently interact with labor market insecurity and housing market conditions, reinforcing cycles of residential disruption. As such, housing instability should be understood not only as a financial or policy issue, but also as a reflection of broader household and social contexts that shape residential stability.

According to the Joint Center for Housing Studies of Harvard University [22], nearly one in four renters is severely cost-burdened, spending more than half of household income on housing. For such families, even minor financial setbacks can quickly destabilize their living situations [23].

Employment instability is another key driver of housing instability, reflecting the cyclical relationship between poverty and housing. Families who lose a source of income often struggle to pay rent, as illustrated by research showing that nearly half of forced moves due to missed payments were linked to income losses [24]. Housing loss, in turn, increases the likelihood of job loss by 11–22%, creating a negative feedback loop that makes stability even harder to regain. Racial disparities further intensify this cycle, as Black and Hispanic families are disproportionately represented in insecure, low-wage employment [25].

The Great Recession of 2008 illustrates the devastating scale of housing instability during economic downturns. Fueled by a housing bubble that expanded rapidly in the early to mid-2000s and relaxed lending practices, many buyers purchased homes they could not afford, often relying on risky loan terms such as zero down payments and very low initial monthly payments [26]. When housing prices fell and favorable loan terms expired, borrowers were left with unaffordable mortgages. Ultimately, more than six million American households lost their homes to foreclosure during this period [27]. This historical episode highlights the vulnerability of families when economic shocks intersect with unstable housing markets.

### 2.2. What Are Adverse Childhood Experiences (ACEs)?

ACEs refer to stressful or traumatic events in childhood that can have long-lasting effects on health and well-being [28]. This framework originated from a landmark study by Kaiser Permanente in the mid-1990s, which shifted attention toward the profound ways early-life experiences shape long-term physical, mental, and emotional health.

Originally, ACEs included various forms of abuse (psychological, physical, sexual) and household dysfunction (e.g., parental substance abuse, mental illness, incarceration, or exposure to domestic violence). Over time, the framework expanded to encompass experiences such as parental separation or divorce, as well as emotional and physical neglect [29].

ACEs are measured through retrospective self-reports, where adults reflect on experiences they had as children. Responses are aggregated into an “ACE score,” with higher scores indicating greater exposure to adversity [30]. Despite decades of research, ACE screening remains limited in many healthcare settings. For example, one study found that only 12.2% of male patients and 25% of female patients were screened in medical contexts [31]. Barriers include lack of time, insufficient training, and low confidence among medical professionals.

The consequences of ACEs are substantial. Research consistently associates higher ACE scores with elevated risks of chronic illness, including heart disease, cancer, respiratory disease, fractures, and liver disease [16]. Moreover, the risks increase cumulatively. As the number of adverse experiences rises, so too does the likelihood of poor physical, mental, and psychosocial outcomes, including depression, substance use, and impaired social functioning. This evidence underscores ACEs as a crucial framework for understanding how early adversity translates into long-term health and social disparities.

### 2.3. Impact of ACEs on Health and Quality of Life

The health impacts of ACEs are mediated by the coping strategies individuals adopt in response to early adversity. Studies have shown a strong link between the number of childhood adversities and the prevalence of adult risk factors, including smoking, obesity, depression, and suicide attempts [16]. ACEs also have intergenerational consequences: parents who experienced high levels of adversity in childhood are more likely to raise children exposed to similar risks [32]. However, this transmission is shaped by mediating factors, and supportive environments, stable housing, economic security, and access to services can reduce the likelihood that adversity persists across generations.

Importantly, research shows that ACEs can affect how the brain develops by changing how genes works, which influences how people handle stress throughout their lives. This cumulative impact highlights the importance of early intervention to prevent long-term harm [33]. Overall, these findings emphasize that ACEs are not isolated events but ongoing determinants of health and quality of life.

### 2.4. How Are ACEs and Housing Instability Related?

The intersection of housing instability and ACEs is complex but often centers on the production of “toxic stress” [34]. Families facing housing instability are exposed to multiple overlapping stressors, such as unemployment, poverty, and food insecurity, that elevate psychological distress. Living in substandard housing adds another layer of strain, especially for low-income households [35]. In such contexts, children’s home, school, and community environments can all become sources of chronic stress [34]. Chronic stress from housing instability is associated with substance abuse, poor mental health, and family conflict, including domestic violence [36].

Frequent moves further exacerbate these challenges by disrupting family routines, weakening social networks, and exposing families to unsafe or resource-poor environments. Stronger social ties have been shown to promote resilience and better health outcomes [35], yet mobility often undermines the ability to form and maintain such ties. Research indicates that high residential mobility in early life is associated with later behavioral problems, suggesting a sensitive developmental period when instability has the most harmful effects [37,38].

Prior research suggests that not all residential mobility carries the same implications for child well-being. In particular, involuntary or financially driven moves are more closely linked to economic hardship, family stress, and disrupted caregiving environments, conditions that heighten children’s risk of adverse experiences. Accordingly, when we conceptualize frequent moves as housing instability, we focus on instability-related mobility pathways rather than residential mobility per se [37]. Such moves often expose families to unsafe or resource-poor environments, thereby increasing the likelihood of ACEs [34,39]. When families live in communities characterized by food insecurity, violence, weak schools, unemployment, and crime, the conditions for ACEs multiply [34], and children may face heightened risk of involvement with child welfare systems.

It is important to note that Moving to Opportunity (MTO) experiment aimed to enhance stability by preventing repeated mobility and resettle families in stable environments. Evidence from the MTO experiment and related studies shows that when families are supported to relocate to safer, lower-poverty neighborhoods, long-term adversity is reduced and outcomes improve [40,41].

While prior studies have revealed important relationships between housing instability and ACEs, their findings are often limited to specific geographic areas. To fully understand the causal pathways and broader implications, national-level analyses are needed. This study builds on existing research by using nationally representative data to examine whether frequent moves increase children’s exposure to ACEs, offering evidence that can inform policy and practice at a larger scale.

## 3. Data and Methodology

### 3.1. Data Description

This study uses data from the National Survey of Children’s Health (NSCH), funded by the Maternal and Child Health Bureau (MCHB) of the Health Resources and Services Administration (HRSA). The NSCH provides nationally representative information on the physical and mental health of children aged 0–17, as well as factors related to family, neighborhood, school, and social contexts.

The survey has been conducted in multiple iterations. Earlier rounds (2003, 2007, 2011/2012) used telephone interviews under the administration of the National Center for Health Statistics. Since 2016, the U.S. Census Bureau has conducted the NSCH annually using mail and web-based methods. In each household, one child is randomly selected, and parents or caregivers respond to the survey on that child’s behalf. Public-use files are available from the Census Bureau.

Our analysis draws on the combined datasets from the 2020 and 2021 survey waves. These datasets were merged using consistent variables across both years, with adjusted sampling weights provided to ensure representativeness of non-institutionalized children at both the national and state levels. The combined dataset includes 93,669 children (42,777 from 2020 and 50,892 from 2021). Of these, 32,860 are aged 0–5, 27,104 are aged 6–11, and 33,705 are aged 12–17. This large sample allows us to capture variation across developmental stages and enhances the generalizability of results.

### 3.2. Variables

#### 3.2.1. Adverse Childhood Experiences (ACEs)

Our primary outcomes are based on parent-reported responses to 11 questions about children’s exposure to ACEs. These include: (1) difficulty affording food or housing; (2) parental divorce or separation; (3) parental death; (4) parental incarceration; (5) exposure to domestic violence at home; (6) exposure to neighborhood violence; (7) living with someone mentally ill, suicidal, or severely depressed; (8) living with someone with drug or alcohol problems; (9) being treated unfairly due to race or ethnicity; (10) being treated unfairly due to a health condition or disability; and (11) being treated unfairly due to sexual orientation or gender identity.

Because not all questions were available in both years or for all ages, we use a consistent subset of nine ACEs. Specifically, items (10) and (11) were excluded because they were either age-limited (sexual orientation/gender identity applies only to ages 6–17) or year-specific (health condition/disability available only in 2021). Responses are coded dichotomously (Yes/No) except for economic hardship, which uses a four-point scale (never, rarely, somewhat often, very often). For economic hardship, responses of “somewhat often” or “very often” are coded as adverse experiences for analysis.

#### 3.2.2. Frequent Moves

The key dependent/independent variable is the number of times a child has moved since birth. Parents or caregivers report this as a numeric value, with responses ranging from 0 (no moves) to 15 (maximum recorded in the survey). In our sample, about 39% of children had never moved, while roughly 10% had moved more than three times. We measure mobility in two ways: (1) a continuous count of total moves, and (2) a dichotomous variable indicating whether the child moved four or more times.

We define frequent moves as four or more residential relocations during childhood. Prior studies have used thresholds ranging from two or more moves within a short time frame to three or four moves across early childhood to identify housing instability [18,42,43,44]. Because the NSCH measure captures cumulative moves since birth, we use a four-move threshold to identify chronic instability over the life course rather than short-term mobility. In our sample, the average number of moves was 1.5, and approximately 88% of children experienced three or fewer moves, while only 12.2% experienced four or more. This threshold therefore captures the upper tail of the mobility distribution and distinguishes sustained instability from more typical residential mobility patterns.

Based on the literature, we hypothesize that, on average, more frequent moves will be associated with higher exposure to ACEs, consistent with prior evidence linking residential mobility to stress, disruption of social ties, and instability.

#### 3.2.3. Metropolitan Residence

We include a measure of whether the household currently resides in a metropolitan area. The influence of urban residence on ACEs is theoretically ambiguous. On the one hand, metropolitan areas provide greater access to healthcare, school, and social services [45,46]. On the other hand, urban environments are also associated with higher exposure to crime, crowding, and certain mental health risks [47,48]. Given these offsetting mechanisms, the expected direction of the effect of metropolitan residence on ACEs is uncertain.

#### 3.2.4. Neighborhood Characteristics

To examine how community environments shape ACE outcomes, we include four measures of neighborhood quality. First, neighborhood support is measured by parent responses to three items: whether people in the neighborhood help each other, watch out for each other’s children, and provide help during times of need. A supportive neighborhood is defined when parents “definitely agree” with at least one item and “somewhat agree” or “Definitely agree” with the others. Prior research shows that supportive neighborhoods and collective efficacy reduce violence exposure and moderate stress-related outcomes [49,50,51]. We therefore expect that children living in supportive neighborhoods will have lower ACE exposure.

Second, neighborhood safety is measured by parent agreement with the statement that “this child is safe in our neighborhood.” Responses are coded dichotomously, where “definitely agree” and “somewhat agree” are classified as safe, and “somewhat disagree” or “definitely disagree” are classified as not safe. Research consistently links perceived neighborhood safety to positive child outcomes, including reduced exposure to violence, lower stress, and greater freedom for healthy social and physical activity [52,53]. Accordingly, we expect that children living in safe neighborhoods will exhibit lower ACE exposure.

Third, neighborhood amenities are measured by the presence of four features: sidewalks or walking paths, parks or playgrounds, recreation or community centers, and libraries or bookmobiles. This categorical variable captures how many of these amenities are present, ranging from 0 (none) to 4 (all). In the regression models, we include a set of dummy variables for one, two, three and four amenities, with “none” as the reference category.

The NSCH provides a limited set of indicators related to neighborhood characteristics. Accordingly, we construct a composite neighborhood amenities measure using all four available items and interpret it as a proxy for cumulative neighborhood resources rather than the independent effect of any single amenity. Sidewalks and walking paths are included as indicators of basic built-environment supports related to walkability and perceived neighborhood conditions, rather than as standalone measures of neighborhood quality.

Neighborhood amenities are often considered protective factors, as access to parks, sidewalks, and community centers provides opportunities for physical activity, social interaction, and engagement with community resources [54,55]. Prior studies show that children in amenity-rich neighborhoods are more likely to have better physical and mental health outcomes due to increased opportunities for safe play, exercise, and parental involvement. Accordingly, we hypothesize that children living in neighborhoods with more amenities will exhibit lower ACE exposure compared to children in neighborhoods with no amenities, though the magnitude of the effect may depend on the number and type of amenities available.

Lastly, neighborhood detracting elements are measured by the presence of three conditions: litter or garbage on the street or sidewalk, poorly kept or rundown housing, and vandalism such as broken windows or graffiti. This categorical variable captures how many of these conditions are present, ranging from 0 (none) to 3 (all), and we include a set of dummy variables for one, two, and three detracting elements, with “none” serving as the baseline category.

Detracting neighborhood features are consistently associated with higher stress, unsafe environments, and negative child outcomes [10,56]. They often signal broader structural disadvantages, including concentrated poverty, weaker social cohesion, and reduced collective efficacy. These environments can limit children’s opportunities for safe play, restrict parental supervision outdoors, and increase exposure to violence and other risks. Therefore, we expect that the presence of more detracting elements will be associated with higher ACE exposure, with greater exposure as the number of such elements increases.

#### 3.2.5. Food or Cash Assistance

To capture the effect of public safety net programs, we include a measure of whether the household received food or cash assistance in the past year. Prior studies suggest that such transfers, including Supplemental Nutrition Assistance Program and other forms of government support, can protect families from severe hardship and reduce the likelihood of eviction or forced moves by easing tradeoffs between food and rent [39,57]. Accordingly, we expect households receiving assistance to be less likely to experience frequent moves.

#### 3.2.6. Parental Work Disruptions

We also incorporate a measure of whether a parent left a job, took a leave of absence, or cut back hours due to the child’s health. Employment disruptions of this kind reduce household income and have been shown to increase the risk of residential mobility and housing instability, particularly when families face additional medical expenses or caregiving demands [58]. Therefore, we anticipate that households with parents who experienced such work disruptions will be more likely to move frequently.

#### 3.2.7. Control Variables

Models of ACEs control for child and family characteristics, including child’s age and sex, child’ race/ethnicity, parental marital status and overall health status, parents’ education, employment, and household income. We also include survey year and state fixed effects to account for time-specific shocks across survey years and unobserved, time-invariant differences across U.S. states, such as variation in housing markets, policy environments, and social service systems, that may influence both residential mobility and children’s exposure to adverse childhood experiences.

#### 3.2.8. Interaction Terms

To assess whether current neighborhood context moderates the association between frequent moves and ACEs, we include two interaction terms: frequent moves × metropolitan residence and frequent moves × supportive neighborhood. These terms allow us to test whether the effect of residential mobility differs depending on urban location or neighborhood support. Because the data capture only current neighborhood characteristics, these interaction terms reflect whether present context conditions the association between past mobility and ACEs, rather than the effects of neighborhood quality at the time of each move.

#### 3.2.9. Descriptive Statistics

In the combined dataset for 2020 and 2021, a total of 93,669 surveys were completed, with 42,777 surveys conducted in 2020 and 50,892 in 2021. Survey responses were weighted according to NSCH sampling procedures to produce estimates representative of the non-institutionalized U.S. child population aged 0–17 residing in housing units both nationally and within individual states. The analytic sample includes 32,860 children aged 0–5, 27,104 children aged 6–11, and 33,705 children aged 12–17. Table 1 provides detailed summary statistics for all study variables.

On average, children experienced 1.50 residential moves, with approximately 12% having moved four or more times. A large majority of children (83%) lived in metropolitan areas, and about 61% resided in neighborhoods rated as supportive. Nearly all children (96%) were reported to live in safe neighborhoods. With respect to community resources, 36% of children had access to all four measured neighborhood amenities (sidewalks, parks, recreation centers, and libraries), while only 11% lived in neighborhoods with none. Conversely, about 24% of children were exposed to at least one detracting neighborhood element, such as litter, poorly kept housing, or vandalism.

ACEs were widely reported. Roughly 10% of children lived in households with economic hardship, 21% had experienced parental divorce or separation, 3% parental death, and 6% parental incarceration. Exposure to domestic violence (5%), neighborhood violence (3%), parental mental health problems (9%), and parental alcohol or drug use (9%) were also evident. Experiences of unfair treatment due to race were reported for 4% of children. Overall, 65% of children had no reported ACEs, 19% had one, 7.5% had two, and 8.4% had three or more.

Control variables indicate that 20% of children lived in households with single parents, and most parents had completed at least some college (62%). About 13% of children lived below the federal poverty line, and 16% in households between 100–199% of poverty. Nearly 9 in 10 parents were employed full-time, with 6% reporting unemployment. The mean child age was 8.7 years, and the sample was evenly split by gender (52% male). In terms of race and ethnicity, 66% of children were White, 14% Hispanic, 7% Black, 6% Asian, and 8% other or multi-racial.

### 3.3. Methodology

Our empirical analysis is based on two model specifications to capture both the determinants and consequences of residential instability. Model 1 examines the predictors of frequent moves, estimating the likelihood and frequency of moving as a function of ACEs and household and neighborhood characteristics:

(1) $$Y_{it}=\beta_{0}+\beta_{\alpha }{ACEs}_{it}+\beta_{x}X_{it}+\epsilon_{it}$$

where the dependent variable, $Y_{it}$, is measured both as a binary outcome (four or more residential moves) and as a count variable. Logistic regression is used for the binary specification, and negative binomial regression for the count specification.

Model 2 then reverses the relationship, testing how frequent moves, urban residence, and neighborhood conditions influence the number of ACEs experienced by children:

(2) $$Y_{it}=\beta_{0}+\beta_{m}M_{it}+\beta_{n}N_{it}+\beta_{u}U_{it}+\beta_{x}X_{it}+\epsilon_{it}$$

where the dependent variable, $Y_{it}$, is the number of ACEs reported by parents of child *i*. The explanatory variable of interest, $M_{it}$, represents the number of moves since birth. $N_{it}$ includes neighborhood characteristics (supportiveness, safety, the number of amenities, and the number of detracting elements). $U_{it}$ captures metropolitan residence, and $X_{it}$ is a vector of child and parent-level controls such as race, age, gender, income, employment status, marital status, and education.

To refine this analysis, our statistical approach involves two steps. First, we estimate separate negative binomial regression models for subsamples defined by frequent moves, urban versus non-urban residence, and interactions between frequent moves and neighborhood characteristics. Because the ACE outcomes are count variables, we use negative binomial regression models. Logistic regression is also applied for dichotomous measures of frequent moves. We also test interactions between frequent moves and neighborhood characteristics, as well as between frequent moves and metropolitan residence, to assess whether supportive environments mitigate the effects of instability.

Second, we distinguish between household-related ACEs (e.g., economic hardship, divorce/separation, death, incarceration, domestic violence, mental health, alcohol/drug) and community-related ACEs (e.g., neighborhood violence, discrimination) by estimating separate regression models for each. This multi-step framework allows us to test different domains of adversity.

Because the NSCH is cross-sectional and does not provide temporal ordering of moves and ACEs, our estimates should be interpreted as associations rather than causal effects.

## 4. Results

Table 2 presents regression results predicting children’s frequent moves, defined as either moving four or more times (logistic regressions, Columns 1–4) or as the total number of moves (negative binomial regression, Column 5). The sequence of models progressively examines the role of cumulative adversity, specific ACEs, economic hardship, household and neighborhood supports, and interactions.

In Column (1), the cumulative number of ACEs is positively and significantly associated with frequent moves. Each additional ACE increases the odds of experiencing four or more moves, highlighting the strong relationship between overall adversity and housing instability.

Column (2) disaggregates the ACEs to identify which experiences drive mobility. All ACE indicators show that all ACEs are positively and significantly associated with frequent moves. Among these, parental divorce or separation emerges as the strongest predictor: children in divorced or separated households have nearly four times the odds of frequent moves (OR = 3.817). Other ACEs such as economic hardship, parental incarceration, and exposure to neighborhood violence also display relatively large effects, reinforcing the strong connection between family instability, community adversity, and residential mobility. While the magnitudes differ, the consistent significance across all ACEs demonstrates the pervasive relationship between childhood adversity and frequent moves.

Column (3) focuses specifically on economic hardship as the core adversity tied to mobility and introduces measures of household and neighborhood supports. Consistent with expectations, hardship significantly increases residential mobility risks. In contrast to expectations, receipt of food or cash assistance is also positively associated with mobility. This pattern likely reflects higher underlying economic vulnerability among households receiving assistance, rather than a destabilizing effect of assistance itself. Work disruptions due to a child’s health condition (“stop or cut work”) are associated with higher mobility, while residence in supportive neighborhoods is associated with a reduced likelihood of frequent moves.

Column (4) incorporates interaction terms to test whether supports moderate the effect of hardship. The food cash × hardship interaction is significant and negative, indicating that the association between hardship and frequent moves is weaker among households receiving food and cash assistance. By contrast, interactions between hardship and supportive neighborhoods or work disruptions are not significant, suggesting these factors operate as direct influences rather than conditional buffers.

Finally, Column (5) estimates a negative binomial regression using the count of moves as the dependent variable. Results are consistent with the logistic regressions, confirming that the findings are not sensitive to the outcome specification.

Overall, these results demonstrate that frequent moves are strongly shaped by household adversity, particularly economic hardship. Yet, targeted supports, especially food and cash assistance, play a meaningful role in reducing housing instability, both by directly lowering mobility and by moderating the risks associated with hardship.

Table 3 presents results from negative binomial regressions estimating the number of ACEs across three categories: total 9 ACEs (Model 1), 7 household ACEs (Model 2), and 2 community ACEs (Model 3).

Frequent moves remain a strong and consistent predictor of adversity. Children who moved frequently experienced substantially higher ACE exposure, with incidence rate ratios (IRRs) of 1.895 for total ACEs, 1.860 for household ACEs, and 2.056 for community ACEs (all *p*-value are less than 0.01).

Neighborhood characteristics show both protective and risk effects. Supportive and safe neighborhoods significantly reduced ACE counts across all models (e.g., IRR = 0.788 for supportive neighborhoods, IRR = 0.841 for safe neighborhoods in total ACEs). By contrast, detracting elements show strong adverse associations: exposure to three detracting elements is associated with a 52% increase in total ACEs and more than double the rate of community ACEs (IRR = 2.404, *p*-value is less than 0.01). Neighborhood amenities provide only modest protection, with significant effects observed at the highest amenity level.

Interaction terms show mixed patterns. The frequent moves × metropolitan interaction reduces community ACEs (IRR = 0.794, *p*-value is less than 0.01), suggesting that urban resources may mitigate mobility risks. Conversely, the frequent moves × supportive neighborhood interaction is unexpectedly positive across all models (e.g., IRR = 1.287 for total ACEs), implying that mobility may disrupt social ties and undermine the benefits of neighborhood support.

Demographic and socioeconomic factors are also influential. Older children have significantly higher ACE counts, with age effects strongest for community ACEs (IRR = 1.110, *p*-value is less than 0.01). Race and ethnicity show distinct patterns: compared to Hispanic children (the baseline), Asian children had significantly fewer ACEs (IRR = 0.583 for total ACEs), while children of other/multi-racial backgrounds have higher ACEs (IRR = 1.201). Black children show lower household ACEs (IRR = 0.858) but higher community ACEs (IRR = 1.670).

Household composition and parental resources are critical. Single-parent households have the strongest association, with children experiencing more than three times the rate of household ACEs (IRR = 3.776, *p*-value is less than 0.01). Parental education above high school is generally associated with higher reported ACEs, especially for those with some college or technical training (IRR = 1.472 for total ACEs). Employment status also matters: children of part-time workers and unemployed parents experience significantly more ACEs, particularly in the community domain. Homeownership is protective, reducing ACE exposure by about 12% (IRR = 0.875, *p*-value is less than 0.01). Poverty gradients are evident: children in households at or above 400% of the federal poverty level have substantially fewer ACEs (IRR = 0.701, *p*-value is less than 0.01) compared to those below the poverty line.

Finally, parental health is strongly associated with children’s ACE exposure. When either parent reports poor health, ACE counts increase by roughly 50%; when both parents report poor health, ACE counts more than doubled for household ACEs (IRR = 2.253, *p*-value is less than 0.01).

Overall, Table 3 shows that frequent moves are a robust predictor of ACEs, but they operate within a broader matrix of family, socioeconomic, and neighborhood factors. While supportive and safe environments offer protection, instability and structural disadvantage magnify risks, particularly in single-parent households and families facing economic hardship or poor parental health.

## 5. Discussion

This study examined the relationship between frequent residential moves and exposure to ACEs using nationally representative data from the 2020–2021 National Survey of Children’s Health (NSCH). The findings demonstrate a robust association between housing instability, measured by frequent moves, and higher rates of ACEs across both household and community domains. Children who experienced frequent moves had more than double the rate of ACEs compared to those in stable housing, reinforcing the strong association between residential instability and adversity and highlighting its relevance to long-term health and social inequalities.

While the associations identified in this study may not be surprising to practitioners working with housing-insecure or child welfare–involved populations, they provide important nationally representative evidence that quantifies the magnitude and persistence of these relationships across household and community contexts. By documenting how frequent residential moves are systematically associated with elevated exposure to adverse childhood experiences, this study underscores that housing instability remains a pervasive and unresolved public health concern, rather than an isolated or anecdotal phenomenon.

Although frequent moves can occur for different reasons, the NSCH does not distinguish between voluntary and involuntary residential relocations. We therefore interpret the strongest observed associations as reflecting instability-related mobility that is more common among economically vulnerable families. At the same time, some households with adequate resources may relocate frequently for opportunity or preference reasons, which could attenuate estimated associations. This heterogeneity reinforces the need for cautious interpretation and highlights the value of future longitudinal research that captures both the timing and reasons for residential moves.

Several broader implications emerge from these findings. First, the results highlight the importance of integrating housing stability into public health and child welfare frameworks. Pediatricians, social workers, and school counselors often assess social and environmental risks; our findings suggest that more systematic attention to housing stability within these existing assessments may further strengthen early identification of children at heightened risk of adversity.

Second, the results highlight the role of neighborhood and community conditions. Metropolitan residence shows a modest protective effect against community-level ACEs, likely reflecting greater access to healthcare, social services, and institutional infrastructure in urban areas. However, this advantage does not extend to household-level adversities, suggesting that neighborhood-level resources alone are insufficient to address deeply rooted household vulnerabilities. Meaningful progress requires sustained public investment in both family-level supports and community infrastructure, along with clear policy prioritization of families experiencing housing instability.

Third, neighborhood characteristics emerge as both protective and conditional. Safe and supportive neighborhoods are associated with significantly lower ACE exposure overall, yet the buffering effect of supportive neighborhoods is not observed among children experiencing frequent mobility. This pattern suggests that residential instability may limit the extent to which families can benefit from neighborhood-level supports, possibly because frequent moves disrupt social ties before they can generate sustained protective effects. However, this finding should be interpreted cautiously. It is also plausible that unobserved household stressors contribute simultaneously to both mobility and ACE exposure, thereby weakening the apparent moderating role of neighborhood context. Moreover, because the data are cross-sectional, we cannot determine the temporal ordering of neighborhood conditions and residential moves. Overall, these interaction results suggest that neighborhood resources may be protective under conditions of stability, but insufficient to fully offset the risks associated with persistent instability.

From a policy perspective, these findings emphasize housing stability as a central social determinant of health and highlight the limitations of existing policy approaches in reducing health inequities linked to housing instability.

Prior research on the social determinants of health has repeatedly emphasized that addressing upstream structural conditions, such as housing stability, is essential for reducing health inequities [59,60,61]. Despite this longstanding evidence, policy responses have often remained fragmented and incremental, focusing on short-term mitigation rather than transformative change. Scholars have noted that this gap reflects not a lack of empirical knowledge, but persistent political and institutional barriers that constrain sustained investment in structurally disadvantaged populations and limit the translation of evidence into policy action [62,63]. The persistence of elevated ACE exposure associated with housing instability documented in this study suggests that, in the absence of coordinated policy action, intergenerational cycles of adversity are likely to continue.

In practical terms, rental assistance, eviction prevention, and policies expanding access to affordable housing are likely to lower the prevalence of destabilizing relocations. At the same time, investments in neighborhood safety, amenities, and social cohesion can reduce exposure to adversity, even when some degree of mobility is unavoidable. Policymakers should also recognize the complex effects of economic hardship, food insecurity, and unstable housing, which magnify the risks of negative outcomes. Integrated policies that address both housing and economic vulnerability may therefore be especially effective.

Despite the strengths of using a large, nationally representative dataset and examining multiple dimensions of ACEs, several limitations should be noted. First, the measure of frequent moves relies on parent or caregiver recall and may be subject to reporting bias. Second, the analysis identifies strong associations but cannot fully establish causal pathways; unobserved factors such as community-level shocks may simultaneously drive mobility and adversity. Third, the NSCH data do not provide temporal sequencing of moves and ACEs, limiting our ability to determine whether moves precede or follow specific adverse experiences. Fourth, measures of neighborhood characteristics are limited to a small set of available indicators and should be interpreted as proxies for cumulative neighborhood resources rather than precise or comprehensive measures of the built environment. Finally, the study period (2020–2021) overlaps with the COVID-19 pandemic, which may have increased both housing instability and ACE exposure, raising questions about generalizability to more stable contexts.

Future research should extend these analyses across multiple years to capture longer-term trends. Longitudinal studies in particular could clarify causality, such as how frequent moves disrupt routines, weaken social networks, and interact with other socioeconomic stressors to shape children’s well-being.

## 6. Conclusions

In conclusion, this study provides strong evidence that frequent moves are associated with heightened exposure to ACEs. From a social justice perspective, these findings suggest that housing instability may function as a structural pathway through which disadvantage and adverse experiences are reproduced across generations, reinforcing health inequities among already marginalized populations. Housing stability is not merely an economic issue but a fundamental determinant of child health and development. Addressing housing instability through combined housing, social, and economic policies is important to interruption cycles of adversity and long-term health problems for children across the United States.

## Figures and Tables

**Table 1:** Descriptive statistics.

	N	Mean	SD	Min	Max
Frequent moves (Count)	91,021	1.500	1.918	0	15
Frequent moves (dummy)	91,021	12.2%	0.328	0	1
Metropolitan	78,585	82.6%	0.379	0	1
Supportive neighborhoods	90,345	60.6%	0.489	0	1
Safe neighborhood	90,849	96.3%	0.189	0	1
Neighborhood amenities	90,400				
None		11.2%	0.315	0	1
1 amenity		11.2%	0.316	0	1
2 amenities		18.5%	0.389	0	1
3 amenities		23.5%	0.424	0	1
4 amenities		35.6%	0.479	0	1
Detracting neighborhood elements	70,718				
None		76.3%	0.425	0	1
1 detracting element		15.0%	0.357	0	1
2 detracting elements		5.1%	0.220	0	1
3 detracting elements		3.6%	0.187	0	1
ACE					
Hardship	91,661	10.0%	0.300	0	1
Divorce/Separation	90,298	21.3%	0.409	0	1
Death	90,154	2.8%	0.164	0	1
Jail	90,067	5.6%	0.230	0	1
Domestic violence	90,011	4.7%	0.2109	0	1
Exposure to violence	89,993	3.3%	0.179	0	1
Mental health	89,965	9.2%	0.289	0	1
Alcohol/Drug	90,010	9.1%	0.287	0	1
Race	89,970	4.0%	0.195	0	1
Number of ACEs	91,902	0.693	1.239	0	9
0		64.5%	0.478	0	1
1		9.4%	0.396	0	1
2		7.6%	0.266	0	1
3+		8.4%	0.278	0	1
Single-headed household	91,060	20.1%	0.401	0	1
Parents’ education	93,669				
Less high school		2.7%	0.163	0	1
High school		13.2%	0.339	0	1
Technical School		21.8%	0.413	0	1
College or high		62.3%	0.485	0	1
Income based on federal poverty level (count)	93,669	285.181	125.500	50	400
0–99% FPL		12.6%	0.332	0	1
100–199% FPL		16.5%	0.371	0	1
200–399% FPL		30.4%	0.460	0	1
Above 400% FPL		40.5%	0.491	0	1
Employment status	90,601				
Full-time		89.6%	0.305	0	1
Part-time		4.0%	0.196	0	1
Unemployed		6.3%	0.244	0	1
Child age	93,669	8.736	5.269	0	17
Child gender (male)	93,669	51.9%	0.500	0	1
Tenure	93,669	81.0%	0.393	0	1
Child race	93,669				
Hispanic		13.6%	0.342	0	1
White, non-Hispanic		66.0%	0.474	0	1
Black, non-Hispanic		6.6%	0.249	0	1
Asian, non-Hispanic		5.6%	0.230	0	1
Other/Multi-racial, non-Hispanic		8.2%	0.275	0	1

**Table 2:** Prediction of frequent moves.

Dependent Variable: Frequent Moves (Binary: 4+ Moves & Count)										
	Odd Ratio		Odd Ratio		Odd Ratio		Odd Ratio		Incident Rate Ratio	
	(1)		(2)		(3)		(4)		(5)	
Total ACEs	1.849	***								
	(0.013)									
Divorce/Separation (d)			3.817	***						
			(0.095)							
Death (d)			1.514	***						
			0.082							
Incarceration (d)			1.578	***						
			(0.063)							
Domestic violence (d)			1.585	***						
			(0.069)							
Neighborhood violence (d)			1.497	***						
			(0.074)							
Mental health (d)			1.578	***						
			(0.053)							
Alcohol/Drug (d)			1.405	***						
			(0.050)							
Discrimination (d)			1.685	***						
			(0.078)							
Economic Hardship (d)			1.739	***	2.342	***	2.846	***	1.674	***
			(0.054)		(0.068)		(0.156)		(0.043)	
Food Cash (d)					1.812	***	1.901	***	1.414	***
					(0.041)		(0.046)		(0.014)	
Stop Cut Work (d)					1.147	***	1.178	***	1.063	***
					(0.045)		(0.057)		(0.021)	
Supportive Nbhd (d)					0.782	***	0.777	***	0.925	***
					(0.017)		(0.019)		(0.009)	
Economic Hardship × Food Cash							0.739	***	0.876	***
							(0.044)		(0.024)	
Economic Hardship × Stop Cut Work							0.925		0.976	
							(0.076)		(0.037)	
Economic Hardship × Supportive Nbhd							1.055		1.005	
							(0.060)		(0.027)	
Year FE	N		N		Y		Y		Y	
State FE	N		N		Y		Y		Y	
Observations	90,068		86,056		88,153		88,153		88,153	
Adjusted R^2^	0.126		0.143		0.053		0.053		0.018	

The regression includes both state and year-fixed effects. *** refers to significance at 1 percent level. Standard errors are given in parentheses.

**Table 3:** Estimation results of the number of ACEs.

Dependent Variable	# ACEs (9)		# HH ACEs (7)		# Com ACEs (2)	
	Incident Rate Ratio (1)		Incident Rate Ratio (2)		Incident Rate Ratio (3)	
Frequent Moves	1.895	***	1.860	***	2.056	***
	(0.064)		(0.062)		(0.167)	
Metropolitan	0.949	***	0.946	***	1.012	
	(0.017)		(0.017)		(0.054)	
Supportive Neighborhood	0.788	***	0.802	***	0.678	***
	(0.011)		(0.012)		(0.026)	
Safe Neighborhood	0.841	***	0.873	***	0.694	***
	(0.021)		(0.023)		(0.034)	
Neighborhood Amenities (1)	0.975		0. 974		1.031	
	(0.023)		(0.024)		(0.066)	
Neighborhood Amenities (2)	0.995		0.990		1.066	
	(0.022)		(0.022)		(0.063)	
Neighborhood Amenities (3)	0.970		0.973		0.987	
	(0.021)		(0.021)		(0.058)	
Neighborhood Amenities (4)	0.952	**	0.951	**	1.028	
	(0.020)		(0.020)		(0.058)	
Neighborhood Detracting Elements (1)	1.135	***	1.120	***	1.256	***
	(0.018)		(0.018)		(0.050)	
Neighborhood Detracting Elements (2)	1.319	***	1.258	***	1.811	***
	(0.031)		(0.030)		(0.093)	
Neighborhood Detracting Elements (3)	1.523	***	1.401	***	2.404	***
	(0.040)		(0.038)		(0.123)	
Frequent Moves × Metropolitan	0.950		0.968		0.794	***
	(0.032)		(0.033)		(0.066)	
Frequent Moves × Supportive Nbhd	1.287	***	1.284	***	1.260	***
	(0.035)		(0.035)		(0.081)	
Age	1.064	***	1.057	***	1.110	***
	(0.001)		(0.001)		(0.004)	
Sex	1.002		0.996		1.024	
	(0.012)		(0.012)		(0.030)	
Race						
White, non-Hispanic	0.990		1.117	***	0.382	***
	(0.018)		(0.020)		(0.017)	
Black, non-Hispanic	0.968		0.858	***	1.670	
	(0.251)		(0.023)		(0.084)	
Asian, non-Hispanic	0.583	***	0.500	***	0.944	
	(0.021)		(0.020)		(0.063)	
Other/Multi-racial	1.201	***	1.138	***	1.518	***
	(0.029)		(0.029)		(0.073)	
Single Parent	3.460	***	3.776	***	1.471	***
	(0.050)		(0.056)		(0.056)	
Education (Less than HS)						
High school degree	1.356	***	1.366	***	1.031	***
	(0.048)		(0.049)		(0.083)	
Some college or technical school	1.472	***	1.466	***	1.295	***
	(0.052)		(0.052)		(0.100)	
College or more	1.230	***	1.199	***	1.332	*
	(0.044)		(0.043)		(0.105)	
Employment						
Part-time employment	1.145	***	1.118	***	1.390	***
	(0.028)		(0.028)		(0.078)	
Unemployed	1.055	**	1.034		1.251	***
	(0.023)		(0.022)		(0.063)	
Tenure	0.875	***	0.864	***	1.013	
	(0.013)		(0.013)		(0.037)	
Poverty Level						
100–199% FPL	1.029		1.027		1.008	
	(0.120)		(0.020)		(0.047)	
200–399% FPL	0.896		0.885	***	0.983	
	(0.017)		(0.017)		(0.047)	
400% FPL or greater	0.701		0.674	***	0.933	
	(0.015)		(0.015)		(0.050)	
Parents health						
Either one is not good	1.521	***	1.514	***	1.497	
	(0.021)		(0.021)		(0.053)	
Both are not good	2.120	***	2.253	***	1.742	
	(0.038)		(0.041)		(0.076)	
Year FE	Y		Y		Y	
State FE	Y		Y		Y	
Observations	69,224		69,224		68,951	
Adjusted R^2^	0.17		0.18		0.17	

The regression includes both state and year-fixed effects. *, **, *** refers to significance at 10, 5, 1 percent level respectively. Standard errors are given in parentheses. Neighborhood Amenities baseline: no amenity; Neighborhood Detracting Elements baseline: no detracting element; Race baseline: Hispanic; Education baseline: Less than HS; Employment baseline: full-time employment; Poverty Level baseline: below 100%; Parents’ health baseline: both are good. “#” denotes the number of ACEs. The dependent variables are the number of total ACEs (0–9), household ACEs (0–7), and community ACEs (0–2).

## Data Availability

The original data presented in the study are openly available from the Data Resource Center for Child & Adolescent Health at https://www.childhealthdata.org/learn-about-the-nsch/NSCH (accessed on 1 December 2025).
